# Human immunodeficiency virus and mortality from coronavirus disease 2019: A systematic review and meta-analysis

**DOI:** 10.4102/sajhivmed.v22i1.1220

**Published:** 2021-04-15

**Authors:** Timotius I. Hariyanto, Jane Rosalind, Kevin Christian, Andree Kurniawan

**Affiliations:** 1 Faculty of Medicine, Pelita Harapan University, Tangerang, Indonesia; 2 Department of Internal Medicine, Faculty of Medicine, Pelita Harapan University, Tangerang, Indonesia

**Keywords:** coronavirus disease 2019, COVID-19, SARS-CoV-2, HIV, AIDS

## Abstract

**Background:**

Persons living with human immunodeficiency virus (PLWH) constitute a vulnerable population in view of their impaired immune status. At this time, the full interaction between HIV and severe acute respiratory syndrome coronavirus 2 (SARS-CoV-2) has been incompletely described.

**Objective:**

The purpose of this study was to explore the impact of HIV and SARS-CoV-2 co-infection on mortality.

**Method:**

We systematically searched PubMed and the Europe PMC databases up to 19 January 2021, using specific keywords related to our aims. All published articles on coronavirus disease 2019 (COVID-19) and HIV were retrieved. The quality of the studies was evaluated using the Newcastle–Ottawa Scale for observational studies. Statistical analysis was performed with Review Manager version 5.4 and Comprehensive Meta-Analysis version 3 software.

**Results:**

A total of 28 studies including 18 255 040 COVID-19 patients were assessed in this meta-analysis. Overall, HIV was associated with a higher mortality from COVID-19 on random-effects modelling {odds ratio [OR] = 1.19 [95% confidence interval (CI) = 1.01–1.39], *p* = 0.03; *I*^2^ = 72%}. Meta-regression confirmed that this association was not influenced by age (*p* = 0.208), CD4 cell count (*p* = 0.353) or the presence of antiretroviral therapy (ART) (*p* = 0.647). Further subgroup analysis indicated that the association was only statistically significant in studies from Africa (OR = 1.13, *p* = 0.004) and the United States (OR = 1.30, *p* = 0.006).

**Conclusion:**

Whilst all persons ought to receive a SARS-CoV-2 vaccine, PLWH should be prioritised to minimise the risk of death because of COVID-19. The presence of HIV should be regarded as an important risk factor for future risk stratification of COVID-19.

## Introduction

At the end of December 2019, the first cases of a newly discovered acute respiratory illness named coronavirus disease 2019 (COVID-19) were reported in Wuhan, China.^1^ By January 2021, >88.3 million infections and 1.9 million deaths worldwide had been reported.^2^ The COVID-19 disease has various clinical manifestations, ranging from mild symptoms such as fever, cough and anosmia to life-threatening conditions including shock, respiratory failure, arrhythmia, overwhelming sepsis and neurological impairment.^3,4^ Meta-analyses have identified several comorbidities,^5,6,7,8,9^ medicines^10,11^ and abnormal laboratory test results^12,13^ associated with a poor outcome. Persons living with human immunodeficiency virus (PLWH) are an at-risk population in view of their impaired immunity. This impairment increases susceptibility to tuberculosis, opportunistic infections and cancer.^14^ In 2019, an estimated 38 million people globally were living with HIV; 1.7 million new (incident) infections and 690 000 deaths were reported that year.^15^ Human immunodeficiency virus–infected individuals with immune suppression (impaired T-cell and humoral responses), unsuppressed HIV RNA viral load (untreated or with treatment failure) and comorbid disease (diabetes mellitus, cardiovascular and renal impairment) may be at risk of the life-threatening forms of severe acute respiratory syndrome coronavirus 2 (SARS-CoV-2) infection.^16^ However, this hypothesis requires additional evidence. Results from observational studies have been conflicting.^17,18,19,20^ This meta-analysis aims to explore the impact of HIV and SARS-CoV-2 co-infection on the mortality outcomes of COVID-19 based on available observational studies.

## Research methods and design

### Eligibility criteria

This is a systematic review and meta-analysis of published observational studies. Articles were selected if they fulfilled the following entry criteria: compliance with the PICO framework, namely P = confirmed positive COVID-19 patients, I = patients living with HIV, C = HIV-uninfected persons and O = mortality in COVID-19-confirmed patients not attributable to unrelated conditions such as trauma. The studies included were randomised clinical trials, cohort, case-cohort and cross-over design, and the full-text paper had to be available and to have been published. Excluded studies included non-original research such as review articles, letters or commentaries; case reports; studies in a language other than English; studies of children and youths <18 years of age and pregnant women.

### Search strategy and study selection

A systematic search of PubMed and Europe PMC provided many papers. Additional articles were located by analysing the papers cited by the authors of the identified studies. The search terms included ‘HIV’ or ‘human immunodeficiency virus’ or ‘immunocompromised’ or ‘immune-deficient’ or ‘AIDS’ or ‘acquired immunodeficiency syndrome’ and ‘SARS-CoV-2’ or ‘coronavirus disease 2019’ or ‘COVID-19’ or ‘novel coronavirus’ or ‘nCoV’. The selected time-range included 01 December 2019 to 19 January 2021. Only English-language articles were evaluated. Details of the search strategy are listed in Table 1. Studies of HIV and SARS-CoV-2 co-infection with a valid definition of ‘mortality’ were included. The search strategy is presented in the preferred reporting items for systematic reviews and meta-analyses (PRISMA) diagram.

**TABLE 1:** Literature search strategy.

Database	Keywords	No. of results
PubMed	(“hiv”[MeSH Terms] OR “hiv”[All Fields]) OR (“acquired immunodeficiency syndrome”[MeSH Terms] OR (“acquired”[All Fields] AND “immunodeficiency”[All Fields] AND “syndrome”[All Fields]) OR “acquired immunodeficiency syndrome”[All Fields] OR “aids”[All Fields]) AND (“COVID-19”[All Fields] OR “COVID-19”[MeSH Terms] OR “COVID-19 Vaccines”[All Fields] OR “COVID-19 Vaccines”[MeSH Terms] OR “COVID-19 serotherapy”[All Fields] OR “COVID-19 Nucleic Acid Testing”[All Fields] OR “covid-19 nucleic acid testing”[MeSH Terms] OR “COVID-19 Serological Testing”[All Fields] OR “covid-19 serological testing”[MeSH Terms] OR “COVID-19 Testing”[All Fields] OR “covid-19 testing”[MeSH Terms] OR “SARS-CoV-2”[All Fields] OR “sars-cov-2”[MeSH Terms] OR “Severe Acute Respiratory Syndrome Coronavirus 2”[All Fields] OR “NCOV”[All Fields] OR “2019 NCOV”[All Fields] OR ((“coronavirus”[MeSH Terms] OR “coronavirus”[All Fields] OR “COV”[All Fields]) AND 2019/11/01[PubDate] : 3000/12/31[PubDate]))	1626
Europe PMC	“HIV” OR “human immunodeficiency virus” OR “immunocompromised” OR “immunodeficient” OR “AIDS” OR “acquired immunodeficiency syndrome” AND “SARS-CoV-2” OR “coronavirus disease 2019” OR “COVID-19”	9107


**FIGURE1:**
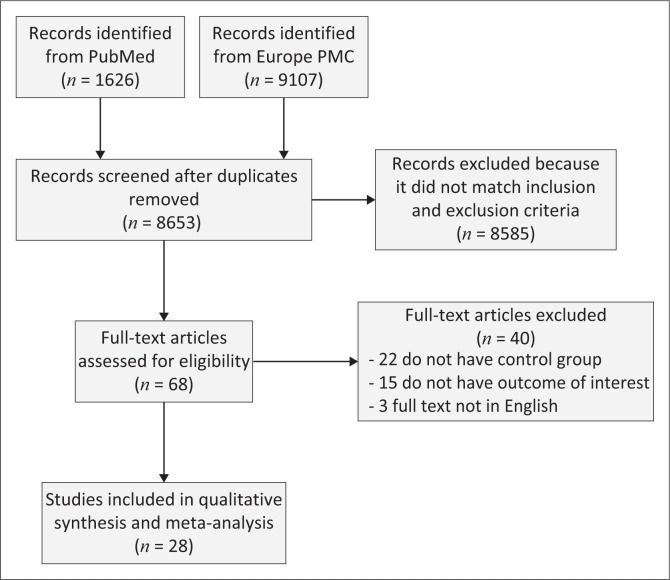
PRISMA diagram of the detailed process of selection of studies for inclusion in the systematic review and meta-analysis.

The initial investigation located 10 733 studies. After the removal of duplicates, 8653 records remained. A further 8585 studies were excluded after screening of the titles and abstracts failed to match with the inclusion and exclusion criteria. Of the 68 full-text articles evaluated for eligibility, 22 that lacked control or comparator groups were excluded, and 15 more were excluded because they lacked outcomes pertinent to our study. Three articles that were not in the English language were rejected. The final meta-analysis included 28 observational studies^21,22,23,24,25,26,27,28,29,30,31,32,33,34,35,36,37,38,39,40,41,42,43,44^ that reported on 18 255 040 COVID-19-infected persons, of whom 48 703 were co-infected with both HIV and SARS-CoV-2 (see Figure 1). Of the included articles, 25 were retrospective and 3 were prospective (see Table 2).

**TABLE 2:** Characteristics of the included studies.

Study	Sample size	Design	Median age, yr (IQR)	Male		Black ethnicity		No. of HIV/AIDS patients:					
				*n*	(*%*)	*n*	(*%*)	Total		CD4 cell counts <200 cells/μL		Receiving ART	
								*n*	(*%*)	*n*	(*%*)	*n*	(%)
Berenguer J et al.^21^ 2020 (Spain)	4035	Retrospective cohort	70 (56–80)	2433	61	12/3915	0.3	26/3962	0.7	N/A	-	21/25	84
Bhaskaran K et al.^17^ 2020 (England)	17 282 905	Retrospective cohort	48 (40–55)	8 632 666	49.9	340 114/17 282 905	1.9	27 480/17 282 905	0.1	N/A	-	N/A	-
Boulle A et al.^22^ 2020 (South Africa)	22 308	Retrospective cohort	52 (37–63)	7052	31.6	N/A	-	3978/22 308	17.8	70/199	35	56/70	80
Braunstein SL et al.^23^ 2020 (USA)	204 422	Retrospective cohort	52 (47–65)	105 024	51.3	32 491/204 422	15.8	2410/204 422	1.1	379/1419	26.7	1288/1447	89
Cabello A et al.^24^ 2020 (Spain)	7061	Retrospective cohort	46 (37–56)	6277	88.9	N/A	-	63/7061	0.9	17/63	26.7	61/63	96.8
Chilimuri S et al.^25^ 2020 (USA)	375	Retrospective cohort	63 (52–72)	236	63	93/375	25	22/375	6	N/A	-	N/A	-
Docherty AB et al.^26^ 2020 (England)	20 133	Prospective cohort	72.9 (58–82)	12 068	59.9	N/A	-	83/20 133	0.5	N/A	-	N/A	-
El-Solh AA et al.^27^ 2020 (USA)	7816	Retrospective cohort	69 (60–74)	7387	94.5	3264/7816	41.7	144/7816	1.8	N/A	-	N/A	-
Garibaldi BT et al.^28^ 2020 (USA)	832	Retrospective cohort	63 (49–75)	443	53.2	336/832	41	9/832	1	N/A	-	N/A	-
Geretti AM et al.^18^ 2020 (England)	47 592	Prospective cohort	74 (60–84)	27 248	57.2	1523/42 320	3.5	122/47 592	0.2	N/A	-	112/122	91.8
Gudipati S et al.^19^ 2020 (USA)	65 271	Prospective cohort	52 (45–67)	30 677	47	20 886/65 271	32	278/65 271	0.4	2/14	14.2	13/14	92.8
Hadi YB et al.^20^ 2020 (USA)	50 167	Retrospective cohort	48 (29–67)	22 636	45.1	12 729/50 167	25.3	404/50 167	0.8	N/A	-	284/404	70.2
Harrison SL et al.^29^ 2020 (USA)	31 461	Retrospective cohort	50 (35–63)	14 306	45.5	8758/31 461	27.8	226/31 461	0.7	N/A	-	N/A	-
Hsu HE et al.^30^ 2020 (USA)	2729	Retrospective cohort	54 (40–68)	1312	48.1	1218/2729	44.6	732/2729	2.7	N/A	-	N/A	-
Huang J et al.^31^ 2020 (China)	50 333	Retrospective cohort	37 (29–52)	5427	90.4	N/A	-	6001/50 333	11.9	613/5897	10.3	5527/6001	92.1
Jassat W et al.^32^ 2020 (South Africa)	41 877	Retrospective cohort	52 (40–63)	19 122	45.6	13 444/19 777	68	3077/35 550	8.7	401/1390	28.8	1271/1278	99.5
Kabarriti R et al.^33^ 2020 (USA)	5902	Retrospective cohort	58 (44–71)	2768	46.9	1935/5902	32.7	92	1.6	N/A	-	N/A	-
Karmen-Tuohy S et al.^34^ 2020 (USA)	63	Retrospective cohort	60 (41–81)	57	90.4	9	14.2	21/63	33.3	6/19	31.5	21/21	100
Kim D et al.^35^ 2020 (USA)	867	Retrospective cohort	57 (46–71)	473	54.7	267/867	30.8	24/867	2.8	N/A	-	N/A	-
Lee SG et al.^36^ 2020 (Korea)	7339	Retrospective cohort	47 (28–66)	2970	40.1	N/A	-	4/7339	0.1	N/A	-	N/A	-
Maciel EL et al.^37^ 2020 (Brazil)	440	Retrospective cohort	53 (42–68)	240	57.1	158/279	56.6	4/440	1	N/A	-	N/A	-
Marcello RK et al.^38^ 2020 (USA)	13 442	Retrospective cohort	52 (39–64)	7481	56	3518/13 442	26.1	159/13 442	2	N/A	-	N/A	-
Miyashita H et al.^39^ 2020 (USA)	8912	Retrospective cohort	55 (42–69)	4922	55.2	N/A	-	161/8912	1.8	N/A	-	N/A	-
Ombajo LA et al.^40^ 2020 (Kenya)	787	Retrospective cohort	43 (33–54)	505	64	N/A	-	53/787	7	N/A	-	N/A	-
Parker A et al.^41^ 2020 (South Africa)	113	Retrospective cohort	48 (34–62)	45	38.9	N/A	-	24/113	21.2	N/A	-	17/24	70.8
Sigel K et al.^42^ 2020 (USA)	493	Retrospective cohort	61 (54–67)	374	75.8	205/493	41.5	88/493	17.8	24/57	42.1	88/88	100
Stoeckle K et al.^43^ 2020 (USA)	120	Retrospective cohort	60 (56–70)	96	80	36/100	36	30/120	25	7/27	25.9	29/30	96.6
Tesoriero JM et al.^44^ 2020 (USA)	377 245	Retrospective cohort	53 (45–67)	51	70.5 vs 50.5	192 646	51	2988/377 245	0.8	270/2887	9.3	2834/2988	94.8

USA, United States of America; ART, antiretroviral therapy; HIV/AIDS, human immunodeficiency virus / acquired immunodeficiency syndrome; IQR, interquartile range; N/A, not applicable.

### Data extraction and quality assessment

The study’s outcome of interest was mortality from COVID-19. This was defined as the number of patients with COVID-19 whose death could not be attributed to a cause other than COVID-19. Two authors performed the data extraction. Relevant demographic, laboratory and clinical information was recorded on a dataform: age, gender, ethnicity, the number of PLWH, the number of patients with a CD4 cell count of <200 cells/μL, the use of antiretroviral therapy (ART) and the mortality outcomes of both HIV-infected and HIV-uninfected participants. Two authors independently assessed the quality of each study using the Newcastle–Ottawa Scale.^45^ The selection, comparability and outcome of each study were assigned a score from zero to nine. Studies with scores of ≥7 were considered to be of good quality (see Table 3). All included studies were rated ‘good’. In summary, all studies were deemed fit to be included in the meta-analysis.

**TABLE 3:** Newcastle–Ottawa quality assessment of observational studies.

First author	year	Study design	Selection	Comparability	Outcome	Total score	Result
Berenguer J et al.^21^	2020	Cohort	***	**	***	8	Good
Bhaskaran K et al.^17^	2020	Cohort	****	**	***	9	Good
Boulle A et al.^22^	2020	Cohort	***	**	***	8	Good
Braunstein SL et al.^23^	2020	Cohort	***	**	***	8	Good
Cabello A et al.^24^	2020	Cohort	***	**	***	8	Good
Chilimuri S et al.^25^	2020	Cohort	***	**	***	8	Good
Docherty AB et al.^26^	2020	Cohort	****	**	***	9	Good
El-Solh AA et al.^27^	2020	Cohort	***	**	***	8	Good
Garibaldi BT et al.^28^	2020	Cohort	****	**	***	9	Good
Geretti AM et al.^18^	2020	Cohort	***	**	***	8	Good
Gudipati S et al.^19^	2020	Cohort	**	**	***	7	Good
Hadi YB et al.^20^	2020	Cohort	**	**	***	7	Good
Harrison SL et al.^29^	2020	Cohort	***	**	***	8	Good
Hsu HE et al.^30^	2020	Cohort	**	**	***	7	Good
Huang J et al.^31^	2020	Cohort	***	**	***	8	Good
Jassat W et al.^32^	2020	Cohort	***	**	***	8	Good
Kabarriti R et al.^33^	2020	Cohort	***	**	***	8	Good
Karmen-Tuohy S et al.^34^	2020	Cohort	**	**	***	7	Good
Kim D et al.^35^	2020	Cohort	***	**	****	9	Good
Lee SG et al.^36^	2020	Cohort	***	**	***	8	Good
Maciel EL et al.^37^	2020	Cohort	**	**	***	7	Good
Marcello RK et al.^38^	2020	Cohort	***	**	***	8	Good
Miyashita H et al.^39^	2020	Cohort	**	**	***	7	Good
Ombajo LA et al.^40^	2020	Cohort	***	**	***	8	Good
Parker A et al.^41^	2020	Cohort	***	**	***	8	Good
Sigel K et al.^42^	2020	Cohort	***	**	***	8	Good
Stoeckle K et al.^43^	2020	Cohort	***	**	***	8	Good
Tesoriero JM et al.^44^	2020	Cohort	**	**	***	7	Good

Note: Asterisk denotes scores.

### Statistical analysis

Review Manager version 5.4 (Cochrane Collaboration) and the Comprehensive Meta-Analysis version 3 software were used in the meta-analysis, and Mantel-Haenszel’s formula gave odds ratios (ORs) and 95% confidence intervals (CIs). The heterogeneity was assessed using the *I*^2^ statistic with values of <25%, 26% – 50% and >50% providing low, moderate and high degrees of heterogeneity, respectively. Significance was obtained if the two-tailed *P*-value was ≤0.05. The qualitative risk of publication bias was assessed using Begg’s funnel plot analysis.

## Results

### HIV and mortality

**FIGURE 2:**
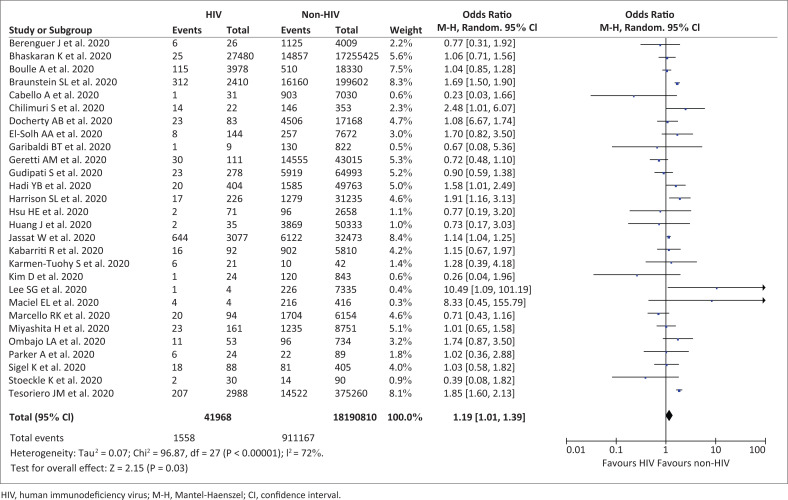
Forest plot that demonstrates the association of HIV with mortality from COVID-19 outcome.

Our pooled analysis indicated that HIV was associated with mortality from COVID-19 [OR = 1.19 (95% CI 1.01–1.39), *p* = 0.03; *I*^2^ = 72%, random-effect modelling] (see Figure 2).

### Meta-regression

**FIGURE 3:**
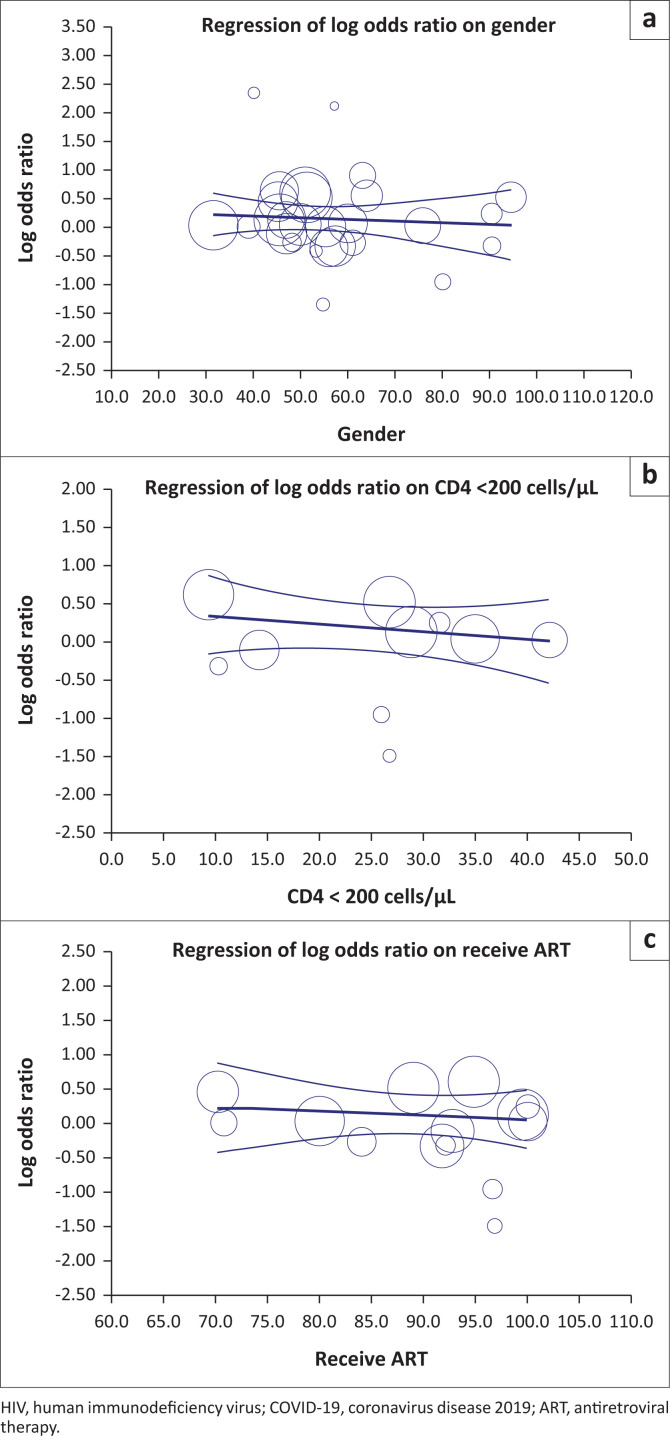
Bubble-plot for meta-regression. Meta-regression analysis showed that the association between HIV and mortality from COVID-19 was not affected by gender (a), CD4 cell count (b) or ART (c).

However, meta-regression showed that the association between HIV and mortality from COVID-19 was unaffected by age (*p* = 0.208), gender (*p* = 0.608) (see Figure 3a), Black ethnicity (*p* = 0.389), CD4 cell count of <200 cells/μL (*p* = 0.353) (see Figure 3b) or ART (*p* = 0.647) (see Figure 3c).

### Subgroup analysis

The subgroup analysis revealed that the association between HIV and mortality from COVID-19 was only statistically significant for studies from African regions [OR = 1.13 (95% CI = 1.04–1.23), *p* = 0.004; *I*^2^ = 0%, random-effect modelling] and the United States of America (USA) [OR = 1.30 (95% CI = 1.08–1.59), *p* = 0.006; *I*^2^ = 61%] but not for studies from Asia [OR = 2.41 (95% CI = 0.16–36.57), *p* = 0.53; *I*^2^ = 76%], or Europe [OR = 0.90 (95% CI = 0.70–1.15), *p* = 0.40; *I*^2^ = 5%].

### Publication bias

**FIGURE 4:**
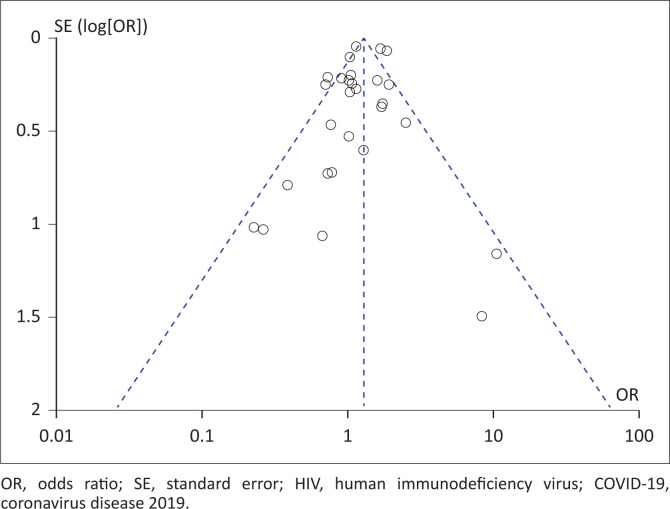
Funnel plot for the association of HIV with mortality from COVID-19 outcomes.

The funnel plot analysis revealed a qualitatively symmetrically inverted funnel plot for the association between HIV and a mortality outcome, suggesting no publication bias. This is demonstrated in Figure 4.

## Discussion

This systematic review and meta-analysis of 28 studies not only analyse the association between HIV and mortality from COVID-19 but evaluate the role of confounding factors such as age, gender, ethnicity, CD4 cell count and ART in this cohort.

An association was found between HIV and mortality from COVID-19. However, this did not appear to be influenced by the confounding factors above. Instead, the subgroup analysis found that mortality from COVID-19 in PLWH was more likely to be reported in studies from Africa and the USA, rather than Asia or Europe. Factors unique to Africa, such as the large background prevalence of HIV, delayed access to healthcare (poor health ‘awareness’, an inadequate healthcare infrastructure and logistical challenges to accessing care) and ready access to alternate, non-Western, traditional health practitioners and medicines, are likely to have influenced outcomes.^46,47^ Similarly, the COVID-19 epidemic in the USA disproportionately affected the poor, people of colour and the socially marginalised such as drug users and the institutionalised. In both regions, PLWH may have been ‘over-represented’ in published studies.

Our pooled data confirmed an association of higher mortality from COVID-19 in PLWH.

Firstly, HIV infection may cause severe depletion of the gut-associated lymphoid tissue, with a predominant loss of memory CD4+ T cells.^48^ Human immunodeficiency virus-induced T-cell lymphopenia, which disrupts the innate and adaptive immune response, may predispose patients to *Mycobacterium tuberculosis* infection and progression to active disease, which increases the risk of latent tuberculosis reactivation by 20-fold.^49,50^ Previously published studies regarding COVID-19 have revealed that the presence of tuberculosis was associated with higher severity and mortality from COVID-19.^51,52^ Secondly, some proportions of PLWH may have incomplete immune reconstitution and evidence of persistent immune activation.^53^ They may show an abnormal innate and adaptive immune response, characterised by the elevation of macrophages, cytokines [tumour necrosis factor alpha, interleukin (IL)-1, IL-6, IL-8 and IL-10], acute phase proteins [serum amyloid A, C-reactive protein (CRP)], elements of the coagulation cascade (D-dimer and tissue factor), increased turnover and exhaustion of T cells, increased turnover of B cells and hyperimmunoglobulinaemia.^54,55^ These conditions may contribute to the development of cytokine storms and severe outcomes in COVID-19. Furthermore, elevated CRP, D-dimer and IL-6 have been associated with severe COVID-19 based on meta-analysis studies.^13,56^ Thirdly, exhaustion of T-cell lymphocytes, which is observed in HIV progression, may also be exacerbated during COVID-19 infection, possibly as a result of the SARS-Cov-2 infection’s synergistic activity with HIV, which gradually results in T-cell lymphocyte apoptosis.^57^ This exhaustion of T-cell lymphocytes was associated with the progression and severe manifestation of COVID-19.^58,59^

### Limitations

Firstly, only a limited number of our included studies reported on CD4 cell counts, viral loads and ART – a fact that is likely to have impacted the precision of the meta-regression analysis of this study. Indeed, most studies focussed on the characteristics of COVID-19 patients rather than its effects on PLWH. Secondly, the studies utilised in this review and meta-analysis were primarily observational and thus, may reflect occult confounders or biases unique to the particular study. Finally, we included some preprint studies to minimise the risk of publication bias; however, we made exhaustive efforts to ensure that only sound studies were included that we expect will eventually be published. We hope that this study can give further insight into the management of COVID-19 patients.

## Conclusion

Our meta-analysis of observational studies indicates that HIV had an association with a mortality outcome from COVID-19; however, larger observational studies or even randomised clinical trials are needed to confirm our results and elucidate additional associations. Patients living with HIV must take extra precautions and always adhere to health-promoting protocols. They must be prioritised to receive COVID-19 preventive therapy: the SARS-CoV-2 vaccine. Where feasible, practical use must be made of telemedicine and virtual-based practice to provide continuous care to PLWH throughout this pandemic. Every effort must be made to identify co-infected PLWH and to link them with clinicians and treatment centres skilled in COVID-19 care. Gaps in ART-related care, such as medicine stockouts, must be identified by local healthcare providers and authorities. Finally, HIV co-infection must be included in future risk stratification models for COVID-19 management.
